# Innate Sex Differences in the Timing of Spring Migration in a Songbird

**DOI:** 10.1371/journal.pone.0031271

**Published:** 2012-02-01

**Authors:** Ivan Maggini, Franz Bairlein

**Affiliations:** Institute of Avian Research “Vogelwarte Helgoland”, Wilhelmshaven, Germany; Pennsylvania State University, United States of America

## Abstract

In migrating animals protandry is the phenomenon whereby males of a species arrive at the breeding grounds earlier than females. In the present study we investigated the proximate causes of protandry in a migratory songbird, the northern wheatear *Oenanthe oenanthe*. Previous experiments with caged birds revealed that males and females show differentiated photoperiod-induced migratory habits. However, it remained open whether protandry would still occur without photoperiodic cues. In this study we kept captive first-year birds under constant photoperiod and environmental conditions in a “common garden” experiment. Male northern wheatears started their spring migratory activity earlier than females, even in the absence of environmental cues. This indicates that protandry in the northern wheatear has an endogenous basis with an innate earlier spring departure of males than females.

## Introduction

In migratory birds, protandry is the earlier arrival of males at the breeding grounds as compared to the females [1]. Different hypotheses (reviewed in [2]) have been formulated in order to explain the evolutionary causes of protandry. Most of them are not mutually exclusive and include direct and indirect fitness correlates. The degree of protandry in birds is an adaptive trait linked to reproductive fitness upon which we may expect selection. Although the ultimate (evolutionary) consequences of protandry have received much attention, knowledge of its proximate causes is still at its beginnings and only a few studies have focused on the mechanisms governing protandry in migratory birds (reviewed in [3]). Based on field observations, four main and not mutually exclusive hypotheses are proposed: (1) males depart earlier from the wintering grounds than females [4], [5]; (2) both sexes have common winter quarters and leave the winter quarters at the same time but males migrate faster than females (e.g. [6]); (3) females overwinter in areas more distant from the breeding grounds than males [7]–[9] and (4) males and females winter in different habitats [4]. Despite these observations it remains open whether protandry has an endogenous component as well. The present study deals with the earlier departure of males from the wintering grounds (hypothesis 1), which is likely to underlie endogenous regulation. Only a few studies were conducted on captive birds [3], [10], [11], all of which revealed an earlier onset of spring migratory activity in males relative to females. However, these studies were conducted either with birds captured in the wild prior to the experiment [3] or with hand-raised birds kept in simulated natural photoperiods [10], [11]. Thus, it cannot be precluded that the differences between males and females in the onset of spring migratory activity were either due to early experience prior to capture or to the photoperiodic cues [12]. Therefore, we aimed to study hand-raised young birds kept under constant photoperiod and otherwise controlled conditions for the whole first year of life because such an experimental design is necessary in order to isolate the intrinsic, endogenous component of protandry. Our model species was the northern wheatear (*Oenanthe oenanthe*), a widespread Holarctic breeding species with all populations overwintering in sub-Saharan Africa [13], which thus represents one of the most stunning migration systems in passerine birds. In the wheatear protandry is reported at breeding grounds [14], [15] as well as at stopover sites during migration [16], [17]. This species has been shown to reveal endogenous migratory restlessness and concurrent migratory fattening [18]. It is therefore a perfect candidate to address the question whether protandry has an innate background.

## Results

We were able to determine the onset of nocturnal activity in spring in 25 out of 31 individuals (12 males and 13 females), and the onset of body mass increase in 37 out of 43 individuals (18 males and 19 females). Table 1 shows the outcome of the GLMMs testing for effects of year, sex and provenance on the onset of nocturnal restlessness and body mass gain in spring. There were effects of year on both variables which were probably due to the different age of the birds at the beginning of the experiments in early autumn. However, this had no influence on the effects of sex on spring behaviour as shown by the outcome of the models (Table 1).

**Table 1:** GLMMs for effects of sex, year and provenance on onset of nocturnal restlessness and body mass increase in spring.

	Onset of nocturnal restlessness			Onset of body mass increase		
Fixed effects	estimate ± SE	z value	P	estimate ± SE	z value	P
Intercept	5.45±0.03	211.43	**<0.001**	5.23±0.03	161.76	**<0.001**
Sex	−0.09±0.03	−2.78	**0.005**	−0.02±0.04	−0.44	0.660
Provenance	0.10±0.03	3.03	**0.002**	0.05±0.04	1.18	0.238
Year	−0.17±0.03	−5.53	**<0.001**	0.12±0.03	3.33	**<0.001**
Prov*Sex				−0.15±0.05	−2.64	**0.008**

Significant effects in bold.

**Figure 1:**
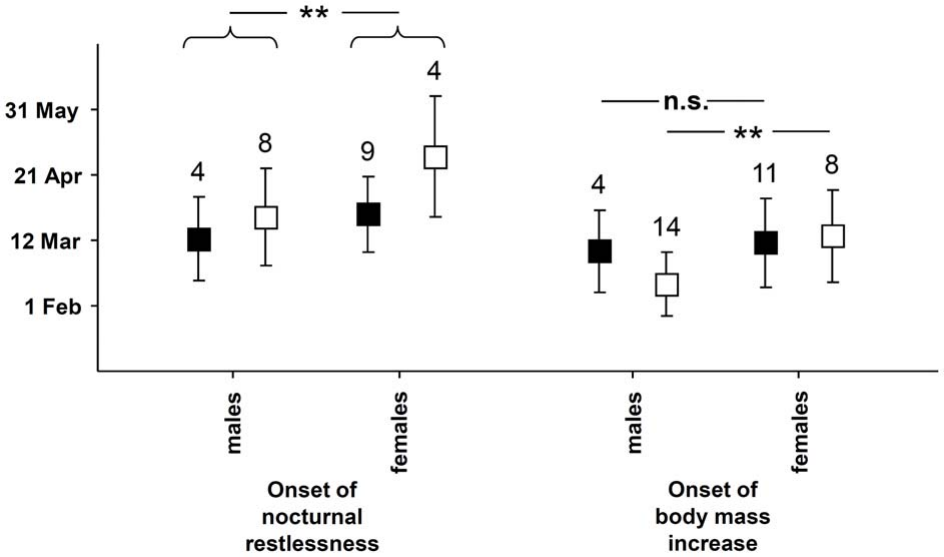
Mean (±s.d.) dates of onset of nocturnal restlessness and of body mass increase in male and female northern wheatears in spring. Black squares: Icelandic population, white squares: Norwegian population. The numbers above each boxplot indicate the sample sizes. Stars indicate *P*-level: **P<0.01. For better understanding, we assigned Day 1 of our count (starting on the day of photoperiodic switch) to a standardized date which was defined as the mean date of photoperiodic switch in the two years of experiment. For our data, this date was 26 August.

In both populations males began their spring nocturnal restlessness on average 17 days (Iceland: 16 days; Norway: 36 days, Figure 1) earlier than females (p = 0.005, Table 1). Additionally, Icelandic birds started their nocturnal restlessness slightly earlier in spring than Norwegian birds (p = 0.002, Table 1).

Regarding the spring onset of body mass increase, males started significantly earlier only in the Norwegian population (p = 0.008, Table 1), on average 29 days earlier than in females (Figure 1). Nevertheless, Icelandic males started increasing their body mass on average 5 days earlier than females, although this difference was not significant (Table 1).

The sex-specific difference in spring migratory behaviour is not a result of different autumn onsets of migratory traits. We were able to determine the onset of nocturnal restlessness in autumn in 14 out of 22 individuals (7 males and 7 females), and the onset of body mass increase in autumn was calculated in all individuals. In autumn there were no differences between sexes in the onset of nocturnal restlessness (Wilcoxon rank-sum test: W = 10.5, p = 0.794). The increase of body mass started on average 5 days earlier in females than males (GLMM: intercept p<0.001; provenance p = 0.003, sex p = 0.021).

## Discussion

Our study shows for the first time that even under constant photoperiodic conditions and without other environmental cues male northern wheatears prepared for and started spring migration earlier than females, thus proving that they are endogenously disposed to depart from their wintering grounds earlier than females.

In general it seems that the degree of protandry was higher in the Norwegian than in the Icelandic population. Populations living further North might respond to completely different time constraints which do not allow a high degree of protandry. This finding supports the theory that the endogenous regulation of migration-related traits, including nocturnal restlessness and body mass changes, is finely regulated and responds to the different needs of different populations [18].

The laboratory observed time lag in onset of spring migratory restlessness between males and females was more pronounced than the observed protandry in wild birds, either at arrival on breeding grounds [14], [15] or during passage at migratory stopover sites [16], [17]. The smaller time lag in wild birds could be attributed to the fine-tuning by photoperiod [19] which was kept constant in our experiment. Additionally, at stopover sites the difference in passage dates may be masked by the passage of different populations with unknown origin and destination. Moreover, other environmental factors may play a role in shaping the innate schedule to the pattern observed in nature, such as adverse weather, predation, parasites, and intra- and interspecific competition during stopover [20].

The observed differences in spring are unlikely to have originated from carry-over effects of the photoperiodic shift in late summer because the onset of nocturnal migratory restlessness did not differ between the sexes. Furthermore, females began increasing their body mass in autumn slightly earlier than males. Therefore, differences observed in spring are based on endogenous mechanisms, possibly due to a different pace of the males' internal clock as compared to females, which might be related to differences in energy turnover between males and females [21].

The finding that males are endogenously pre-disposed for earlier spring departure from wintering sites has important implications for a better understanding of the consequences of climate driven phenological changes in migratory birds [22]. Phenological traits can evolve quickly within just a few generations when the selective pressures are high [23]. A response to strong selection may cause considerable changes in the patterns of sex-differentiated timing of migration, and ultimately impact on the reproductive success of the species [3].

## Materials and Methods

### Ethical Note

All animals were handled according to international ethical standards. Licences for capture were granted from the Ministry for the Environment (Iceland, licence nr. UMH05030044/13-4-1 HS/– and UMH06040104/13-4-1 of 12 May 2006) and the Directorate for Nature Management (Norway, licence nr. 05/3039 ART-VI-ARES of 28 April 2005 and 2005/3039 ART-VI-ARES of 3 May 2006). The Institute of Avian Research has a general permission for housing birds from the Landesregierung Niedersachsen (LAVES: 509f-42502-32/12 of 30 July 2004), and no additional permissions are required in Germany for behavioural studies. After the experiment, the birds were kept in captivity for further behavioural studies.

### Study birds and methods

Nestling northern wheatears were taken from their nests at an age of 5–8 days. We took 16 birds from Iceland (5 males and 11 females) and 27 birds from Norway (15 males and 12 females). Birds were taken from both sites in 2005 and 2006 (see Supporting Information Table S1 for details). After being taken from their nests, birds were transported to the Institute of Avian Research in Wilhelmshaven, Germany, within 2–3 days, and fed by hand with mealworms until they were able to feed independently. At this point, their diet constituted a mix of live mealworms and a standardized food containing dried insects and other additives [24]. Birds were kept singly in cages of 50×40×40 cm and were housed in two identical rooms with no windows and a constant temperature of 20°C±2°C. Males and females from both populations were randomly positioned in both rooms. The photoperiod was kept constant at 14L∶10D until the birds were approximately 45 (in 2006) to 75 (in 2005) days old, and was then switched to 12L∶12D. The day count started on the day of photoperiodic switch, and our analyses are based on this count.

The activity of the wheatears was automatically recorded by highly sensitive microphones (Piezo-Scheibe 27 mm, Conrad Electronic SE, Hirschau, Germany) attached to the cage walls. Impulses were collected into a device (developed by R. Nagel, Wilhelmshaven, and S.F. Becker, Bremen, Germany) which created a CSV-file containing the counts of the movements of every bird, summarized over 15-minute intervals. We took the number of 15-minute intervals with activity per night as a unit for quantifying the amount of nocturnal restlessness [25]. The first and last hour of the night were not considered, so that every night consisted of 40 intervals (10 hours). Nocturnal restlessness was measured in 31 birds in total (Iceland: 5 males and 11 females; Norway: 9 males and 6 females). In addition to recording nocturnal activity, body mass changes were tracked along the whole experimental season, since body mass changes are closely linked to migration [26]. Weighing was carried out twice a week, always in the morning, just after the lights in the rooms went on. The measurements were stopped around mid-May in the following spring.

The onset of spring migratory nocturnal restlessness was defined as the first day after 1 February when the activity was constantly over a threshold of four 15-minute intervals per night (see Supporting Information S1). The onset of body mass increase in spring was determined by calculating mean body mass in 5-day periods, and taking the first period when mean body mass was increasing for at least five following periods. The exact date of onset was then defined to be the third day in the 5-day period determined as above. To assess whether the differences in timing in spring were due to carry-over effects of autumnal events we determined the dates of onset of nocturnal restlessness and body mass increase in autumn, using the same procedure as described above. In the first year the measurement of nocturnal restlessness started only later in the season, where most birds had already begun their nocturnal activity, due to technical problems. Therefore, the comparison of the onsets of nocturnal restlessness in autumn was made only between males and females tested in the second year.

We tested for differences between sexes in onset of nocturnal restlessness and body mass increase in spring and autumn, including provenance, year, and the interactions between all terms as fixed effects, and nest as a random effect, in a general linear mixed model (GLMM) with a Poisson error structure. We simplified the models by stepwise eliminating non-significant terms until reaching a best-fit model according to the principle of maximum parsimony. Due to the smaller sample size, we were not able to perform a GLMM for testing for differences in the beginning of nocturnal restlessness in autumn. Therefore, we compared the dates of onset for males and females with a Wilcoxon rank-sum test. Statistical testing was performed using the software R 2.8.0.

## Supporting Information

Supporting Information S1 Supplementary methods. Provenance of test birds, estimation of onset of nocturnal restlessness and threshold selection.
(DOC)

Table S1 Dates and site of collection, sample sizes and date of photoperiodic switch for our study birds.
(DOCX)
