# Supplementary material for: Innate Sex Differences in the Timing of Spring Migration in a Songbird
Source: PLoS One. 2012 Feb 1;7(2):e31271. doi: 10.1371/journal.pone.0031271 (PMC3270037; doi:10.1371/journal.pone.0031271)
Supplement: Supporting Information S1 — Supplementary methods. Provenance of test birds, estimation of onset of nocturnal restlessness and threshold selection. (DOC) [file pone.0031271.s001.doc]

**SUPPORTING INFORMATION S1**

To the article**: “Innate sex differences in the timing of spring migration in a songbird”** (Ivan Maggini and Franz Bairlein)

**Supplementary Methods**

Provenance of test birds

**Table S1**. Dates and site of collection, sample sizes and date of photoperiodic switch for our study birds.

| Population | Year | Site | Date collected from nest | *N* | | Date of photoperiodic switch |
| --- | --- | --- | --- | --- | --- | --- |
|  |  |  |  | ♂ | ♀ |  |
| Iceland | 2005 | Myvatn Lake^1^ | 29 June | 2 | 6 | 6 September |
| Iceland | 2006 | Öxnadalðalur^2^ | 3-7 July | 3 | 5 | 15 August |
| Norway | 2005 | Rogaland^3^ | 26-27 June | 8 | 5 | 6 September |
| Norway | 2006 | Norddalen^4^ | 3 July | 7 | 7 | 15 August |

^1^Coord.: 65°39’N, 16°36’W

^2^Coord.: 65°37’N, 18°29’W, distance from previous site approximately 85 km

^3^Coord.: 58°43’N, 6°12’E

^4^Coord.: 60°51’N, 6°11’E, distance from previous site approximately 230 km

Estimation of onset of nocturnal restlessness

Note: the method described below is the same used in [S1] and is published in the Electronic Supplementary Material of this reference.

Because nocturnal activity may show irregular patterns which vary between individuals [S2], we needed to develop a method which made it possible to estimate dates of onset of nocturnal restlessness in a comparable way. The method presented below is applicable in the same fashion to birds with regular activity, which show activity values over the threshold during the whole active time, as to birds whose activity value fluctuates around the threshold more often throughout the season. The examples are based on real data. We first created a graph representing the mean activity counts in 5-day periods (Figure S1 as an example).

To estimate the period where the date of onset lies, we applied the following criteria:

1. there must be at least two consecutive periods with a value over the threshold, which we define as Periods 1* and 2* (Figure S1);


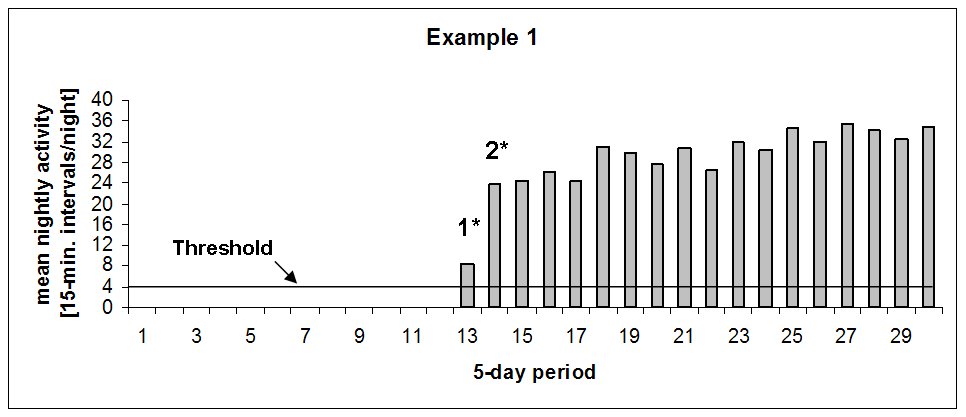


**Figure S1** Mean activity counts in 5-day periods of a bird showing constant nocturnal activity over the season. The date of onset of nocturnal restlessness is in the first period with a mean above the threshold (Period 1*).

1. in the 10 periods following Period 1* (Periods 2* to 11*), the activity value must not be under the threshold more than twice;
2. if this is the case (as e.g. Figure S2), the procedure must be repeated from the next two consecutive periods with mean activity over the threshold, which then become Periods 1* and 2*;


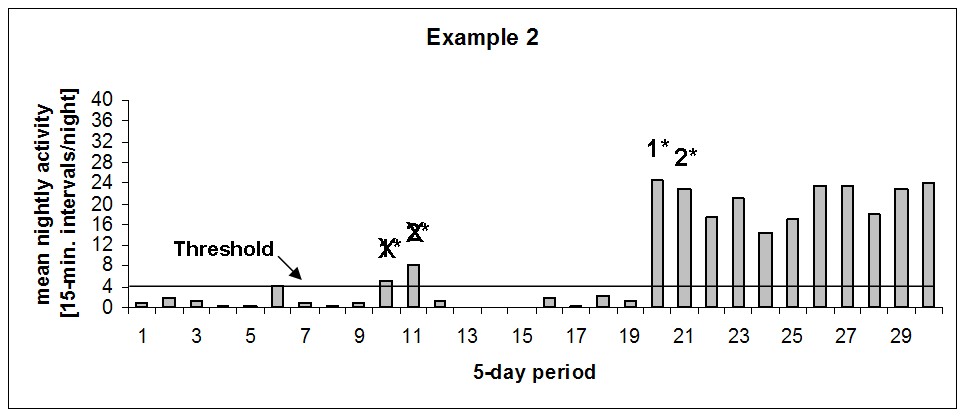


**Figure S2** Mean activity counts in 5-day periods of a bird showing irregular nocturnal activity over the season. The date of onset of nocturnal restlessness is not in the first period with a mean above the threshold, but later in the season (Period 1*, see text).

1. in case the conditions are never satisfied, the date of onset is not calculated. The same applies in cases where the mean activity in a 5-day period exceeds the threshold in five periods or less during the whole season.

After having estimated Period 1*, the exact date of onset was calculated as follows:

1. create a graph with the activity counts of the single nights in Period 1* and the previous period (defined as Period -1*, Figure S3 as an example), for a total of 10 nights;
2. if the activity count exceeded the threshold for two consecutive nights, the first of the two was taken as date of onset of nocturnal restlessness.
3. if during the whole 10 nights there were never two subsequent nights with activity over the threshold, the date of onset was set on the last (tenth) night of the sequence.


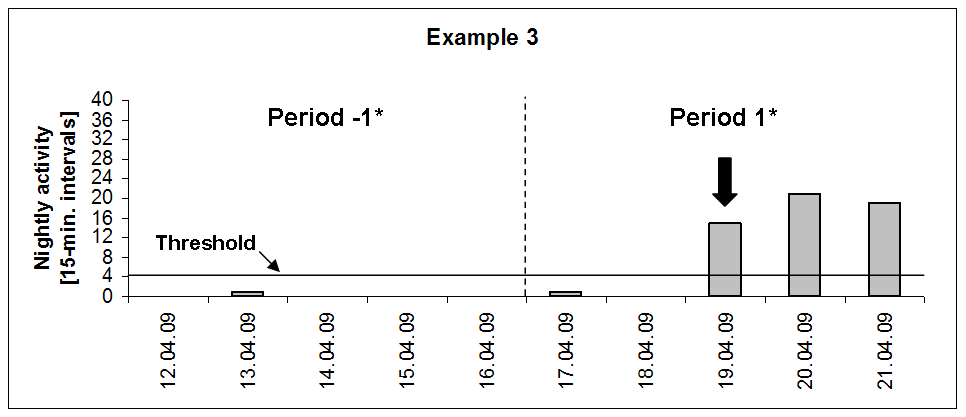


**Figure S3** Activity counts in the single nights during Periods -1* and 1*. The arrow shows the estimated date of onset of nocturnal restlessness, which is the first of two subsequent nights with activity values over the threshold (see text).

Threshold selection

Very small amounts of nocturnal activity might not be directly linked to nocturnal restlessness. For this reason we needed to choose a threshold value which might best represent a night with actual migratory restlessness. For this sake we estimated the date of beginning of nocturnal restlessness for every bird using the abovementioned method with different threshold values, and then chose the minimum threshold value after which the result remained constant. There were no birds for which this value was more than 4. Since this represents 10% of a single night (at least one hour with activity), and because it had been used in previous publications [S1], we decided to use this value for this analysis as well. Additionally, testing all data using a threshold of 2 did not substantially change the results (the differences between sexes in onset of spring nocturnal restlessness and body mass increase were maintained and still significant).

**References**

S1. Maggini I, Bairlein F (2010) Endogenous rhythms of seasonal migratory body mass changes and nocturnal restlessness in different populations of northern wheatears *Oenanthe oenanthe*. J Biol Rhythms 25: 268-276.

S2. Schindler J, Berthold P, Bairlein F (1981) Über den Einfluß simulierter Wetterbedingungen auf das endogene Zugzeitprogramm der Gartengrasmücke *Sylvia borin*. Vogelwarte 31: 33-44.
