# Supplementary material for: Innate Sex Differences in the Timing of Spring Migration in a Songbird
Source: PLoS One. 2012 Feb 1;7(2):e31271. doi: 10.1371/journal.pone.0031271 (PMC3270037; doi:10.1371/journal.pone.0031271)
Supplement: Table S1 — Dates and site of collection, sample sizes and date of photoperiodic switch for our study birds. (DOCX) [file pone.0031271.s002.docx]

| Population | Year | Site | Date collected from nest | *N* | | Date of photoperiodic switch |
| --- | --- | --- | --- | --- | --- | --- |
|  |  |  |  | ♂ | ♀ |  |
| Iceland | 2005 | Myvatn Lake^1^ | 29 June | 2 | 6 | 6 September |
| Iceland | 2006 | Öxnadalðalur^2^ | 3-7 July | 3 | 5 | 15 August |
| Norway | 2005 | Rogaland^3^ | 26-27 June | 8 | 5 | 6 September |
| Norway | 2006 | Norddalen^4^ | 3 July | 7 | 7 | 15 August |

^1^Coord.: 65°39’N, 16°36’W

^2^Coord.: 65°37’N, 18°29’W, distance from previous site approximately 85 km

^3^Coord.: 58°43’N, 6°12’E

^4^Coord.: 60°51’N, 6°11’E, distance from previous site approximately 230 km
